# The Application of Single-Cell RNA Sequencing in Vaccinology

**DOI:** 10.1155/2020/8624963

**Published:** 2020-08-06

**Authors:** Andrés Noé, Tamsin N. Cargill, Carolyn M. Nielsen, Andrew J. C. Russell, Eleanor Barnes

**Affiliations:** ^1^The Jenner Institute, University of Oxford, Old Road Campus Research Building, Oxford OX3 7DQ, UK; ^2^Peter Medawar Building for Pathogen Research and Oxford NIHR Biomedical Research Centre, Nuffield Department of Medicine, University of Oxford, South Parks Road, Oxford OX1 3SY, UK; ^3^Translational Gastroenterology Unit, John Radcliffe Hospital, Oxford OX3 9DU, UK; ^4^Wellcome Sanger Institute, Wellcome Genome Campus, Cambridge CB10 1SA, UK

## Abstract

Single-cell RNA sequencing allows highly detailed profiling of cellular immune responses from limited-volume samples, advancing prospects of a new era of systems immunology. The power of single-cell RNA sequencing offers various opportunities to decipher the immune response to infectious diseases and vaccines. Here, we describe the potential uses of single-cell RNA sequencing methods in prophylactic vaccine development, concentrating on infectious diseases including COVID-19. Using examples from several diseases, we review how single-cell RNA sequencing has been used to evaluate the immunological response to different vaccine platforms and regimens. By highlighting published and unpublished single-cell RNA sequencing studies relevant to vaccinology, we discuss some general considerations how the field could be enriched with the widespread adoption of this technology.

## 1. Introduction

Vaccines are one of the most effective public health interventions in history and have been extremely successful in preventing illness and death from many infections. Much of this success can be attributed to the discovery of disease-causing agents and/or by the discovery of how to cultivate these pathogens to allow large-scale production of attenuated vaccines. While it is clear that effective vaccines induce protective immunological memory, the precise mechanisms by which this manifests are often poorly understood. Moreover, there are many diseases against which we have not developed successful vaccines, often a result of not fully understanding the “ideal” immune response and/or how to induce this with vaccination. Currently used techniques, such as ELISAs, ELISpots, flow cytometry, and growth inhibition assays, broadly measure responses in the T cell or humoral compartments after vaccination, but cannot agnostically measure differences in response between single immune cells [1–3]. Single-cell RNA sequencing (scRNA-seq) is a relatively novel tool which provides the advantage of understanding responses to vaccination at the level of the individual cell in an unbiased manner.

RNA sequencing quantitatively profiles the cellular transcriptome. Polyadenylated messenger RNA (mRNA) molecules are often the target as the polyA tail is a convenient handle to selectively target the protein-coding mRNA (as opposed to other RNA types). In bulk RNA-seq studies, many thousand cells may be pooled together, obscuring heterogeneity. scRNA-seq (in contrast to bulk) allows the dissection of previously unappreciated levels of heterogeneity. This is a major motivation for embarking in scRNA-seq studies [4, 5]. Over 25 scRNA-seq techniques have been developed in just over a decade, all essentially following five steps: (1) single cell isolation, (2) cell lysis and RNA capture, (3) RNA reverse transcription to cDNA, (4) cDNA amplification, and (5) pooling and sequencing using library preparation, pooling, and next-generation sequencing techniques [5]. Some of the most used scRNA-seq techniques include Smart-seq2 [6], MARS-seq [7], 10x Genomics Chromium [8], inDrop [9], and Seq-Well [10]. The precise differences between these techniques have been discussed extensively by Kolodziejczyk and colleagues [11], with the major differences relating to the resulting transcript data (including sensitivity, accuracy, and transcript portion profiled), throughput, single-cell isolation method, and sequencing platform.

The relative paucity of published reports of single-cell transcriptomic responses in the context of vaccination suggests that there remains much to be learned from scRNA-seq. As with all new techniques, there are difficulties in establishing robust, scalable, and cost-effective protocols for the generation and analysis of scRNA-seq data [12]. However, these obstacles are countered by the opportunity to elucidate complex networks of cell interactions and immune responses and the potential to identify novel or unanticipated response profiles, which have been beyond the scope of bulk RNA and other sequencing technologies. scRNA-seq can serve as the backbone for several other “omics” technologies, where the transcriptome can be profiled in the same cell as well as surface proteins (CITE-seq and REAP-Seq) [13, 14], chromatin accessibility (ATAC-seq) [15], and genomes (G&T-seq and DR-seq) [16–18]. The combination of these technologies allows new subpopulations to be revealed, which would not otherwise be possible by the use of each alone [19, 20], although in-depth discussion of these technologies is beyond the scope of this review.

This review considers the applications of scRNA-seq in prophylactic vaccine development, with a focus on infectious diseases. We use examples from several diseases to demonstrate the flexibility of the technology. We explore published and unpublished literature to highlight existing applications of this technology and provide recommendations and predictions as to how vaccinology could be enriched with its widespread adoption. To illustrate the adaptability of scRNA-seq, we present the case study of COVID-19 vaccine development and discuss the contribution unbiased transcriptional profiling could make.

## 2. Profiling Immune Responses to Infections

Our understanding of the mechanisms underlying immune responses in health and disease has important implications for vaccine design. Previously, targeted techniques have allowed us insights into specific parts of the immunological system during development, during infection, and after infection. scRNA-seq allows the immune system to be studied in an unbiased manner. Additionally, studying single cells allows quantitation of the heterogeneity in systems and to resolve time during dynamic processes. Studying the immune response to infection can provide a window to understanding the challenges that must be overcome by vaccination. This is particularly relevant in diseases such as influenza or malaria where natural infection does not engender complete protection [21, 22]. Here, we highlight uses of scRNA-seq for profiling different components of the immune response in the context of natural or artificial infection, as well as concurrent sequencing of pathogen and host, and responses in the specific context of controlled human infection studies.

### 2.1. Innate and Adaptive Responses to Pathogens

The particular innate cell types and pathways that trigger an effective adaptive immune response have been the focus of recent work by Blecher-Gonen et al. The authors used scRNA-seq to characterise the initial 48 hours of the cellular response to several fluorescently labelled inactivated pathogens [23]. As early as 24 hours after immunisation, relatively rare antigen-carrying cells showed pathogen-restricted programs of transcription. Fluorescent antigen-positive neutrophil and monocyte populations were found almost exclusively in inactivated Mycobacteria-immunised mice, whereas antigen-positive macrophages were mainly found in inactivated Candida-immunised mice. This analysis elucidates initial pathways after inactivated vaccine administration and shows that scRNA-seq can disentangle populations that vary widely in lineage, activation status, and antigen uptake. Further work is needed to identify whether these different innate pathways do in fact correlate with natural or vaccine-induced protection. If they did, regimens could include adjuvants and use specific platforms to trigger the type of innate activation that has been identified to be protective. Indeed, scRNA-seq would be a well-suited tool for this subsequent work.

Long-lived plasma cells are crucial to maintaining high levels of antibodies long after infection and vaccination [24]. Lam et al. prospectively sorted subsets of plasma cells formed in response to natural infections in mice and performed scRNA-seq to define plasma cell transcriptional heterogeneity [25]. In keeping with previous reports [26], approximately a third of plasma cell transcriptomes were made up of kappa light chain constant region transcripts. Despite there being significant metabolic differences in the subsets of plasma cells, metabolic state did not appear to correlate with transcriptional profile. Genes crucial for B cell longevity, including CD28, BLIMP-1, and B-cell maturation antigen, were not differentially expressed across plasma cells, regardless of longevity. There were no stable changes in transcription between long- and short-lived plasma cell subsets. Despite glucose uptake being shown to be important for plasma cell longevity and it being known that transcriptional changes are essential for the establishment of metabolic programs in plasmablasts [27], transcription does not seem to distinguish mature plasma cell subsets further. Indeed, a multiomic tool may assist in resolving these subsets—differences may be accounted for at the protein level by translational control. Analogous studies also need to be performed in humans to determine whether plasma cells generated in response to natural infection and vaccination behave similarly and whether further transcriptional investigation is necessary.

Peripherally circulating CD8+ T cells have been associated with immune control of HIV [28]. Elite controllers of HIV are people who, in the absence of antiretroviral therapy, suppress viral replication (in contrast to chronic progressors). Nguyen et al. compared the characteristics of HIV-tetramer-specific CD8+ T cells in the blood and lymph nodes of elite controllers and chronic progressors [29]. Weakly cytolytic CD8+ T cells preferentially homed to B cell follicles and vigorously suppressed replication of HIV in elite controller lymph nodes. These CD8+ T cells upregulated expression of numerous soluble factors and cytokines and downregulated inhibitory receptors. The authors argued that these features identified a CD8+ protective immune signature in EC. These results provide guidance for vaccine design, towards the type of CD8+ T cell signature that may be necessary for effective vaccine-induced protection.

Memory CD4+ T cells are required for long-lived immunity and are induced by vaccination strategies, including against malaria and influenza [30, 31]. Additionally, influenza-specific CD4+ T cells correlate with protection against influenza challenge in humans [32]. To this end, Ciucci et al. profiled the transcriptional programs driving CD4+ T cell heterogeneity and memory T cell development [33]. The authors performed iterative scRNA-seq profiling of total T cells, CD4+ T cells, and virus-specific CD4+ T cells at seven days post lymphocytic choriomeningitis virus infection in mice. By comparing the gene expression differences in clusters, Ciucci et al. identified a specific T cell memory precursor signature. The establishment of the memory precursor signature and generation of a long-lived CD4+ response required the transcription factor Thpok. Knowledge of this signature and the mechanistic importance of Thpok in early CD4+ T cell memory could be used to predict longer term CD4+ responses induced by vaccination and, potentially, the generation or lack of vaccine-induced protection.

Rato and colleagues used scRNA-seq to investigate CD4+ T cell heterogeneity prior to HIV infection [34]. To explore the basis of CD4+ T cell permissiveness to HIV infection, they examined for the growth, and infection-permissiveness, of primary cells by a pseudotyped HIV-based vector. The main factor determining transcriptional heterogeneity was the degree of response to TCR stimulation, or cellular activation, which, in turn, manifested in varying degrees of HIV permissiveness. “HIV-permissive cells” identified prior to infection allowed discovery of a gene signature that divided populations into high- and low-permissive subsets. In a similar study, single-cell viral RNA quantitation was performed to demonstrate the correlation between cell gene expression and HIV latency [35]. Analogous immunoprofiling analyses could be performed in the context of vaccinated individuals to determine whether HIV-permissive or HIV-resistant cells are enriched and whether these populations correlate with vaccine-induced protection.

### 2.2. Dual scRNA-seq of Pathogen and Host

scRNA-seq can be deployed to simultaneously interrogate both pathogen and host transcriptomes. Transcriptional profiling at high resolution has enabled an in-depth appreciation of the cellular diversity in biological organisms and the number of transcriptional states during infection. This can allow the interpretation of immune responses to intracellular pathogens at single-cell resolution, as bulk isolates are often heterogeneous [36]. Host-pathogen scRNA-seq has been comprehensively reviewed by Penaranda and Hung [37], but close consideration of how dual scRNA-seq, the simultaneous scRNA-seq analysis of a pathogen and its infected host, can be leveraged for specific vaccinology uses is warranted.

Several groups have recently used dual scRNA-seq to profile virally infected cells and draw insights from transcriptome information [38–42]. O'Neal et al. revealed the feasibility and value of West Nile Virus- (WNV-) inclusive scRNA-seq as a method for single-cell transcriptomics and WNV RNA detection [38]. There was extreme heterogeneity in viral quantity and antiviral gene expression among *in vitro* infected cells. The expression of IFN-stimulated genes in single cells negatively correlated with intracellular viral RNA abundance. Selecting vaccine adjuvants or platforms to generate IFN-stimulated gene products may therefore be a promising WNV vaccine approach. Similarly, Russell and colleagues profile host and influenza virus mRNAs from a variety of cell lines early after *in vitro* infection [39]. They demonstrate astonishing differences in the transcriptional load resulting from influenza infection between ostensibly identical cells in spite of a relatively pure influenza inoculum. These types of approaches could be implemented *ex vivo* in the case of human challenge-compatible pathogens such as influenza to provide insights into the cellular characteristics associated with vaccine-induced protective immunity.

### 2.3. Human Challenge Studies

Responses to infection can also be interrogated in a more regulated setting using controlled human infection models. These involve the direct inoculation of an infectious agent in order to evaluate the subsequent immune response and/or potential protective efficacy of interventions. Barton et al. have already discussed the use of transcriptomics in controlled human infection models [43]. Here, we discuss specific cases where scRNA-seq could be leveraged for particular benefit in vaccine development.

In the context of malaria, Tran et al. set out to profile the differences in the bulk blood transcriptome of challenge-protected and challenge-nonprotected volunteers during and after malaria immunisation [44]. The authors found robust transcriptomic changes, four weeks after immunisation, that were unique to protected volunteers. These changes included T cell, NK cell, protein synthesis, and mitochondrial signatures. The authors detected similar signatures three weeks after controlled human malaria infection (CHMI) and hypothesised that ongoing T cell memory response and clearance of antigen was driving this signal and mediating protection from malaria. scRNA-seq could extend these analyses by characterising immune subsets associated with protection and by identifying TCR clones, across time points, that share enrichment of hypothesised protective pathways to determine if clones are maintained and if there is a clonal dominance in protected individuals.

Mpina and colleagues assessed variations in NK, NKT, and MAIT cell populations using samples from a CHMI study of Tanzanian adults challenged with *P. falciparum* parasites [45]. CHMI decreased MAIT cell frequencies during blood-stage malaria and was followed by a “rebound” increase in circulating MAIT frequency for up to 168 days post CHMI. These cells showed distinct single-cell RNA expression profiles at each time point suggesting that MAIT cells respond to sporozoite challenge by day 9 and do not return to baseline transcriptional in the time period examined. Although MAIT cells express an invariant *α* chain, there are differences in the CDR3 junctions that allow tracking of cell clones. Interestingly, there were no changes in the relative distribution of MAIT cell clones by day 28 after CHMI. This paper demonstrates that the utility of scRNA-seq is not only restricted to interrogating the adaptive immune response. Innate-like and innate cellular responses are likely critical, and underappreciated, to adjuvant and vaccine efficacy [46], as such scRNA-seq is a tool that can unlock insights from human challenge models.

It is clear that scRNA-seq could provide valuable insights for vaccine redesign and targeting in the context of controlled human infection models. Indeed, owing to the relatively common use of these models in malaria vaccine development, most of the published analyses relate to this pathogen. Investigation of other pathogens used in challenge models, such as influenza, *Salmonella typhi*, and BCG [47–49], will likely benefit from scRNA-seq adoption. There is likely to be a vast number of blood samples that have already been obtained in these types of studies, on which it is not possible to perform scRNA-seq, for example, blood sampled in PAXgene Blood RNA Tubes or bulk RNA extracted from peripheral blood mononuclear cells (PBMC). CIBERSORTx [50], MuSIC [51], Bseq-SC [52], and deconvSeq [53] are tools that could be used to deconvolute bulk RNA-seq data derived from these samples. Use of CIBERSORT [54] suggests that *P. vivax* CHMI induces potent immunosuppression mediated by dendritic cells and that this immunosuppression conditions subsequent antimalarial immunity [55]. While these tools are incredibly useful in situations where bulk RNA has been isolated, they function by using known single-cell transcriptional signatures and do not serve as discovery tools for new pathways and cellular heterogeneity. Thus, there is importance of implementing and comprehension of scRNA-seq techniques by vaccinologists.

## 3. Vaccine Evaluation

The evaluation of the immune response to vaccination in both the preclinical and clinical phases is central to the prediction of success in disease protection. Antibody titres are correlates of protection for many, if not most, vaccines and vaccine candidates [62]. These can be measured with minimal sample preparation and equipment, and as a result, we rely heavily on titres as correlates of protection. Antibodies, however, are not always sufficient for protection. Cellular immunity can kill or suppress intracellular pathogens and may synergise with antibodies. T cell responses are indispensable and probably understudied as correlates of protection. Assessment of B and T cell clonality induced by vaccines, vaccine-induced cell phenotypes, and transcriptional signatures associated with protection are all important avenues of investigation that can be achieved through scRNA-seq. Here, we focus on examples of scRNA-seq used as a tool for comparison of vaccination routes and regimens, applications for BCR and TCR analyses, and also comment on cancer vaccines.

### 3.1. Comparing Vaccine Regimens and Responses

Immunogenicity of vaccines is modulated by a number of factors including vaccine antigen, vaccine platform, and adjuvant. scRNA-seq allows the impacts of these to be investigated with a high degree of specificity. scRNA-seq can also be used to characterise the heterogeneity in response to different vaccine regimens. Sheerin et al. performed comparative transcriptomics of the response to the capsular group B meningococcal vaccine (4CMenB), administered concomitantly with other vaccines and on its own, and the response to its constituent antigens or several comparator antigens in mice ([63]; Table 1). The authors found neutrophil-specific genes were enriched at 24 hours following 4CMenB vaccination using bulk RNA-seq. To resolve whether concomitant immunisation resulted in differential transcriptional activity of neutrophils, scRNA-seq was performed on neutrophils isolated from 4CMenB + routine vaccines or routine vaccines-only immunised groups. Here, scRNA-seq has been used to characterise the specific innate immune receptor genes involved in different vaccine regimens providing mechanistic insight into differences in reactogenicity.

Vaccine human challenge studies (VHCS), a subset of controlled human infection models discussed above, involve the direct evaluation of vaccine efficacy by administration of an infectious agent to human volunteers after vaccination. Since the inception of VHCS such as those by Theodore Woodward in the 1940s [64], the means to evaluate vaccines have expanded past vaccine efficacy, clinical symptoms, and antibody titres. To date, however, there have been no published studies utilising scRNA-seq in the context of VHCS. This technique could be used to dissect the heterogeneity of cellular responses in participants with full, partial, or absent vaccine-induced protection after infectious challenge. Additionally, monitoring alterations in the single-cell gene expression profile of the challenge microbe itself could significantly improve understanding of host-pathogen interactions. Dunachie et al. performed whole transcriptome profiling of two groups receiving different malaria vaccine combinations [65]. The transcriptomes of stimulated PBMCs from three participants fully protected from malaria showed enrichment of modules associated with IFN induction and antigen presentation. This was conserved across vaccine regimens and suggests a common vaccine-induced protective pathway. scRNA-seq could extend these analyses by dissection of the particular cell types that contribute to these modules and their relative contributions.

In addition to effects modulated by vaccine regimen, platform, and immunisation route, there is ample evidence to suggest that gene expression following immunisation can be affected by adjuvant selection. Transcriptomic evaluation of nonhuman primates (NHP) and human responses to vaccine adjuvants, with and without vaccination, has largely been restricted to bulk and/or microarray analyses [66–69]. Using an NHP model to compare responses against the HIV Env antigen with eight different adjuvants, Francica et al. [70] demonstrated using microarrays with whole blood RNA that TLR4 or 7 agonists had differential effects on the upregulation of inflammatory and IFN genes. Furthermore, these gene signatures correlated across all adjuvant groups with antibody Fc functionality. Such comparative adjuvant studies in NHPs can support downselection of candidates for translation to humans and provide better targeting of pathogen-specific immune responses. There are thus opportunities for scRNA-seq approaches to further our understanding of adjuvant mode of action, which remains limited, and its association with immunogenicity and/or efficacy. Specifically, combining single-cell technologies with serial tissue sampling may allow superior insight into immune cell activation at the site of immunisation and in the draining lymph nodes, events which can be difficult to capture using other technologies.

### 3.2. BCR and TCR Analyses

For many pathogens, our understanding of protective epitopes is incomplete. In the study of B cells, peptide arrays and phage displays are methods that have been used for the discovery of linear epitopes [71, 72]. The search for T cell targets has frequently focused on HLA-A∗02-restricted epitopes, prevalent in individuals of Caucasian ancestry [73]. Epitope discovery relies on the identification of short peptide sequences within the longer peptide product of the pathogen that evoke an immune response. This can be determined experimentally using functional assays such as interferon gamma ELISpot to identify T cell responsiveness to different peptide sequences or computationally using peptide binding prediction algorithms (reviewed in [74, 75]).

scRNA-seq methods have the potential to improve antigen screening and selection, by providing a more accurate picture of the immune response generated by vaccines with different antigenic make-ups. The development of algorithms that reconstruct T cell receptor sequences from single-cell data allows parallel analysis of the T cell transcriptome and TCR clonotype in multimer sorted antigen specific cells [76, 77]. Redmond et al. describe single-cell TCRseq, a bioinformatic pipeline which identifies mRNA reads that align to genes coding for the variable (V) and constant (C) regions of the T cell receptor (TCR), to reconstruct paired alpha and beta TCRs. Eltahla et al. describe an alternative computational method, VDJ puzzle, to reconstruct TCR sequences from scRNA-seq data. They show that this method can identify TCR clones that are not detected by single-cell PCR of the same sample and can link this analysis to the transcriptome of a cell. These algorithms can be paired with vaccine-reactive cell identification by fluorescent probes conjugated to vaccine antigens. For example, vaccine-reactive T cells were identified following a single dose of a yellow fever vaccine and were characterised by scRNA-seq with TCR sequence linkage [78].

Methods using the transcriptome to identify vaccine-responsive T cells without knowing their epitope specificity have also been developed, which allows a broad understanding of the T cell repertoire in response to vaccination. Fuchs et al. used scRNA-seq to identify a genetic signature of virus-responsive cells by performing scRNA-seq on dye-labelled antigen-specific cells [79]. They identified key genes that are differentially expressed in virus-responsive cells compared to unresponsive cells and show the combined expression of TNFRSF9, XCL1, XCL2, and CRTAM can be used to identify antigen-specific T cells from an undifferentiated T cell population. This enables identification of antigen-responsive cells without the need to sort populations by fluorochrome-labelled multimer prior to sequencing. Further studies of vaccine responses in controlled trials could provide better evidence upon which to select the most immunogenic vaccine epitopes.

Upon antigen stimulation, B and T cells proliferate and undergo clonal expansion; the BCR or TCR sequences are effectively a “clonal barcode.” This can provide information on antigen specificity and cell ancestry. A great strength of scRNA-seq is the ability to obtain unbiased transcriptome and V (D) J gene transcript usage information from the same cell. With the advent of new scRNA-seq workflows, a proliferation of bioinformatics tools to analyse these data has necessarily occurred. BALDR, an example of such a bioinformatic pipeline, is able to reconstruct the paired heavy and light chain immunoglobulin gene sequences from scRNA-seq data derived from Illumina short reads ([80]; Table 1). Upadhyay and colleagues applied BALDR to single plasmablasts, splenic germinal centre B cells, and memory B cells following vaccination of rhesus macaques with an HIV vaccine. The reconstruction accuracy was 100% for the plasmablasts and >80% for the other cell populations, demonstrating that even in rhesus macaques, a species with poor annotation of the immunoglobulin loci, BALDR can recreate paired antibody sequences. This tool has broad applications in vaccinology and has already been used in the preclinical assessment of HIV vaccines ([81]; Table 1).

In a similar fashion, TraCeR [82] and other tools [76–78] allow for the reconstruction of full-length, paired T cell receptor sequences from T cell scRNA-seq data. These methods, however, are limited by either their reliance on the plate-based Smart-seq2 method, Illumina short-read sequencing, the need for a large number of sequencing reads [83], or a failure to integrate switching diversity and alternative mRNA splicing involving the 3′ end of immunoglobulin heavy chain mRNA [84]. Repertoire and Gene Expression by Sequencing (RAGE-Seq) provides a means to sequence full-length antigen receptor transcripts with Oxford Nanopore sequencing and link this with short-read transcriptome profiling at single-cell level [85]. This can also be applied to droplet-based scRNA-Seq workflows. As the availability of commercial assays to analyse antigen receptors—such as 10x Genomics V (D) J library preparation kits—becomes more available, cost-effective, and user-friendly, antigen specificity and ancestry of T and B cells following will be more effectively understood.

In the context of dengue virus (DENV) vaccination, approaches centred solely on B cell-mediated protection have limitations [86]. CD4+ T cells are required to generate and maintain B cell responses and CD8+ T cells are critical for eliminating infected cells. Waickman et al. use scRNA-seq to assess the diversity and long-lived nature of CD8+ T cell responses to an experimental tetravalent DENV vaccine ([87]; Table 1). The investigators followed T cell responses from acute activation to memory time points and compared the overlap of TCR clonotypes. By looking at the occurrence of T cells with an expanded TCR clonotype and by enumerating the presence of these TCRs at late time points, the authors were able to identify a memory precursor subset of CD8+ T cells. These memory precursors had enrichment of cellular metabolism and proliferation gene pathways. Waickman and colleagues demonstrate the ability of scRNA-seq as a tool to accurately and longitudinally track vaccine-antigen-specific T cells across time to identify correlates of T cell-mediated immunity with single-cell resolution. Importantly, scRNA-seq is used in combination with flow cytometry as means for both validation of discovered markers and further hypothesis testing.

Using scRNA-seq data, it is possible to predict cell trajectories, that is, to computationally order cells along putative trajectories, by inferring how much progress an individual cell has made through a given process (such as cell differentiation). The above analyses by Waickman et al. could be extended by using pseudotime tools such as Monocle [88], Slingshot [89], or others (which are benchmarked by Saelens et al. [90]) to infer a trajectory of T cell differentiation. One could then infer as to whether further differentiation is correlated with shared or “public” TCR clones. RNA velocity [91], a high-dimensional vector predicting the future state of an individual cell at a scale of hours, could also be used to infer the directionality of these cell state progression trajectories.

Pairing TCR sequence and transcriptome information allows the discovery and exploration of new cell populations. Afik and colleagues performed scRNA-seq on Yellow Fever Virus (YFV) vaccine-reactive and other CD8+ T cells ([78]; Table 1). Unexpectedly, YFV-specific cells were found across two clusters: one containing effector memory CD8+ T cells and one containing naive CD8+ T cells, thus defining “naïve-like” and “effector memory-like” YFV-specific CD8+ T cells. Combined TCR-transcriptome analysis revealed that the CDR3 sequence was longer in naive-like compared to memory-like cells for both alpha and beta TCR chain. In addition, the authors compared scRNA-seq transcript abundance to protein level by reviewing index sort flow cytometry data, demonstrating the utility of coupling scRNA-seq with other omics technologies. This is an example of how the heterogeneity in the T cell compartment can be investigated following vaccination and accounted for by cell state and TCR properties.

### 3.3. Evaluating Vaccination in High-Risk Populations

Immunocompromised individuals are at risk of higher acquisition and complication rates of many vaccine-preventable infectious diseases such as seasonal influenza, respiratory syncytial virus (RSV), and bacterial pneumonia. In parallel, immunological responses to vaccination are often less efficient compared to healthy adults [98–101].

Optimising vaccine immunogenicity in immunosuppressed populations is paramount, but heterogeneous underlying mechanisms of immunosuppression make this challenging. Causes of altered immune states range from pathological conditions (including primary immune deficiencies and/or acquisition of chronic viral infection with HIV or cytomegalovirus (CMV)) to physiological states (including neonates, pregnant, and older persons) and iatrogenic immunosuppression following organ transplant or treatment for autoimmune conditions. With respect to pathological conditions, coinfections may further complicate vaccination, for example, HIV with hepatitis B or hepatitis C virus.

Study of the immune response at the cellular level in conditions of immunosuppression has demonstrated the nuances of vaccination responses. For example, the contribution of humoral and cellular responses to both influenza and RSV vaccination is altered in older compared to younger adults [102–105]. Contrastingly, CMV infection is associated with attenuated vaccine responses across age groups [106–108]. scRNA-seq is ideally placed to discover how these observations fit together, by profiling heterogeneous cell populations in detail. Clusters of cellular functional networks can be identified, which may in turn uncover key transcriptional drivers that can be targeted to optimise vaccine responses. scRNA-seq could be utilised in the study of alternative vaccine regimens in the context of high-risk populations, to evaluate changes in immunogenicity. For example, adjuvant and increased dose influenza vaccines have been trialled in multiple settings of immunosuppression including older persons and transplant recipients (whose immunosuppressive regimen can vary) [98]. Detailed immunological profiling has not been conducted comparing, head to head, the response to a given vaccine in these different groups. scRNA-seq would be especially useful at discovering common signatures of immunologically successful vaccination regimens, especially in less common settings such as organ transplantation.

### 3.4. Personalised Cancer Vaccines

Cancer vaccines are different from those protecting against infectious diseases in many ways; most notably that they can be used in therapeutic and personalised capacities [109]. Samples from tumour biopsies or resections are composed of a variety of cell types including tumour, immune, and mesenchymal cells. This assortment of cell types can confound bulk RNA-seq data. Single-cell techniques can tackle this challenge.

Single-cell transcriptomic profiling can be incorporated into neoepitope selection and vaccine manufacture workflows. Petti and colleagues performed matched whole-genome sequencing and droplet-based scRNA-seq on samples from patients with acute myeloid leukaemia [110]. They were able to discriminate between tumour and wild-type cells, identify abnormally differentiated tumour cells, and discover mutation-associated transcriptional profiles. In doing so, the authors identified surface markers that could be used to purify and analyse subclones for downstream studies. The approach developed in their paper could theoretically be applied to any cancer type. In this case, scRNA-seq can narrow personalised cancer vaccine workflows to mutations of expressed genes. This approach, albeit using bulk RNA-seq, has already gained traction in the field of melanoma vaccines [111, 112]. CITE-seq uses scRNA-seq in addition to oligonucleotide-labelled antibodies to allow simultaneous protein and transcriptome measurement at a single-cell level [13]. This technique may further enhance neoantigen discovery pipelines, by providing insight into protein expression given dynamic ranges of transcription [113].

Recognising that therapeutic responses are varied in pathologically identical tumours and even in genetically homogeneous cancer cells [114], scRNA-seq provides the ability to discern which cells respond favourably to vaccines. Single-cell sequencing has already been used in the optimisation of targeted drug treatment against metastatic renal cell carcinoma [115]. The authors' approach was able to identify the most effective drug combination for multiple subpopulations of tumour cells. Such approaches could potentially be used in patient xenograft models to determine the most effective and personalised vaccine combinations. Ott et al. analysed the transcriptome of neoantigen-reactive CD4+ single T cells using HLA class II tetramers before and after vaccination [112]. The main gene expression changes reflected transitions from naïve to effector and memory states. These changes included repression of genes promoting homeostatic survival of naïve T cells and upregulation of genes involved in Th1 fate polarisation. Therefore, implementation of scRNA-seq in evaluation of cancer vaccine responses is feasible and has already begun.

Single-cell sequencing must overcome a number of challenges prior to its wholesale adoption in the field of cancer vaccines. These hurdles are present in many scRNA-seq experiments but have specific consequences in cancer vaccine discovery. A major difficulty is “drop-out.” This happens when a transcript or an allele in a heterozygous mutation is not captured or amplified and can occur at 10–50% of mutation sites [5, 116]. While there are computational models that can correct for this drop-out [117], this is of critical importance in cancer vaccinology as it may be impossible to determine whether a cell is truly wild type for a given mutation. Partial transcript coverage is confined to end-biased platforms such as the 10x Genomics Chromium platform. Coverage decreases nonlinearly across the length of a transcript, so some variants are much more easily detectable than others. The utility of scRNA-seq to detect expressed mutations is therefore dependent on the specific mutational composition of the tumour and sample in question. As a result, scRNA-seq will likely perform better in mutation detection for cancers with high mutation burdens [110]. The increase in throughput of techniques such as G&T-seq, allowing simultaneous transcriptomic and genomic profiling from single cells, could potentially circumvent some of these hurdles to scRNA-seq adoption in cancer vaccine workflows.

## 4. Challenges Associated with the Uptake of scRNA-seq

The wholesale adoption of scRNA-seq in vaccinology is limited by hurdles relating to analysis, technical issues, and the experimental questions that can be asked with the technology (see Appendix A for further considerations). For further discussion of the challenges in driving scRNA-seq and other single-cell techniques forward, we direct you to the review by Lähnemann and colleagues [128].

While there is increasing development of computational tools and reference databases [129, 130], it will become progressively more difficult to compare studies based on primary analyses of raw data. It is impractical to suggest that a single analysis pipeline could satisfy the needs of all primary analyses; however, there is a huge potential for studies performing secondary analyses on multiple published raw datasets. This is of particular relevance when considering comparisons of vaccine regimens, screening for correlates of vaccine-induced protection, and evaluating adjuvants. This will necessitate open data access and thorough annotation of experimental methods and parameters.

There are several challenges related to the scRNA-seq technology itself. Every scRNA-seq protocol begins with the preparation of a single-cell suspension of the tissue of interest. When making inferences from scRNA-seq experiments, there are two inherent assumptions related to this step. These are that (i) the cellular composition of the suspension is a faithful representation of the original tissue and (ii) sample preparation results in insignificant (or no) transcriptional changes. Enzymatic, as well as mechanical, dissociation can result in biases of cellular representation as certain cells may be more sensitive to enzymes or dissociation [131]. The latter assumption has also been challenged [132]. Several sources have suggested that isolation of nuclei and their use for single-nucleotide RNA-seq could help to mitigate both of these challenges [131, 133, 134] (see Appendix A).

By beginning reverse transcription using a poly (T)-oligonucleotide, the majority of scRNA-seq technologies use the mRNA polyA tail to synthesise the first strand of cDNA. Challenges with this approach include an inability to capture nonpolyadenylated microRNAs and regulatory RNAs [135], although techniques have been developed to overcome these hurdles [136]. This is of particular relevance in vaccinology as miRNAs are important in B cell antibody affinity maturation, may modulate infection susceptibility and vaccine responses, and can be used as immune modulators and adjuvants [137]. Additionally, scRNA-seq is estimated to detect 30% of mRNA molecules present in a cell, while other approaches such as seq-FISH detect >80% of targeted transcripts in situ, with recent iterations sure to increase this coverage [20, 138]. However, seq-FISH can be difficult to adopt in nonspecialist laboratories due to the microfluidic and microscopic expertise needed. In time, improvements in the sensitivity of conventional scRNA-seq methods and/or increased usability of in-situ hybridisation based scRNA-seq methods will undoubtedly lead to greater insights.

Ultimately, most cell-cell and extracellular cell-pathogen interaction is protein-mediated. For scRNA-seq, inferences about cell-cell interactions occurring between receptor-ligand pairs can be made using repositories of ligands, receptors, and their interactions, such as CellPhoneDB v2.0 [139]. This, and other methods [140], can be used to characterise communication among cells in homeostatic as well as pathological conditions. In many scRNA-seq studies, the transcriptome is used as an index of protein expression; however, several discrepancies limit comparisons that can be made (see Figure 2 in [141]). For example, the differences in half-lives of mRNA and the proteins they encode, intracellular trafficking, and posttranslational modifications all have the potential to alter the function of cells that ostensibly have comparable mRNA levels [141, 142]. Technologies such as CITE-seq, REAP-seq, and Ab-seq complement restrictions of solely RNA-based analyses. Combination techniques also present us with an opportunity to understand the dynamics of these systems as we can take advantage of the very different half-lives of the measured molecules; RNA levels can change at a timescale of seconds, while proteins can persist for days. Furthermore, this highlights scRNA-seq as a high-yield adjunct to be used in conjunction with more conventional vaccinology techniques, rather than a panacea.

The spatial position of cells in tissues strongly influences function, yet there remains no truly single-cell, unbiased, spatial transcriptomics approach. Several approaches, however, are reaching cellular resolution (e.g., Slide-seq with 10 *μ*m voxels) and/or are extremely highly multiplexed (e.g., SeqFISH+ which can measure up to 10,000 genes in single cells) [143–146]. The coming single-cell spatial transcriptomic approaches may be of particular use in the evaluation of vaccines that are mediated by site-specific immunity, for example, through lung tissue-resident memory T cells following tuberculosis vaccination [147].

## 5. Conclusion and Recommendations

Profiling the immune response to both natural and artificial pathogen exposure by scRNA-seq has advanced our ability to identify favourable immunological profiles. The capability of scRNA-seq to concurrently examine the global gene expression, antigen-specificity, clonality, and individual copy number variants (CNVs) and infer the developmental trajectory of immune cells offers a powerful toolbox to appraise host responses to vaccine candidates. Certain areas have not yet been tackled by scRNA-seq, including critical confounders of immunogenicity such as coinfections and age, vaccine platforms, and adjuvants. In addition, we were unable to find any studies primarily using scRNA-seq to better understand adverse events following vaccination, or any other vaccine safety metrics, with the exception of one study discussed above [63].

scRNA-seq is now a widespread research tool; the number of diseases and areas of research in which it is being applied is growing. Improvements in the scale of adoption, robustness and ease of use of reagents, instrumentation, and computational tools mean that scRNA-seq will continue to be used more. The utility of this tool in vaccine design and development is contingent on the particular questions which are being asked (see Appendix A).

Beyond the anticipated improvements in system efficiencies and the increased availability of reagents, what is the best way that scRNA-seq can be applied systematically in vaccine design, development, and evaluation? This will depend on specific hypothesis-driven, experimental, and analytical considerations. In Box 3, we present the specific case study of COVID-19 vaccine development to demonstrate the potential of this technology, and in Box 4, we illustrate more general applications of scRNA-seq in vaccine development and evaluation. Ultimately, scRNA-seq, and its integration with other single-cell systems, will elucidate further information that can be used to drive favourable responses following therapeutic and/or prophylactic vaccination. The context provided by information from classic measurements, such as growth inhibition assays, antibody levels, and cytokine secretion, and controlled human infection studies can further enhance the value of the observations that are made. The generation of integrated datasets is computationally intensive but can provide comprehensive characterisations of vaccine responses by increasing the power of statistical calculations or capturing a greater amount of heterogeneity in systems. These insights can be used to identify novel populations, previously unappreciated correlates, and biomarkers of favourable and protective immune responses for use in the systematic and streamlined assessment of vaccine candidates.

## Figures and Tables

**Figure 1:**
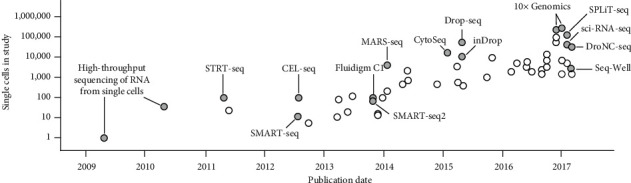
scRNA-seq technologies that have been critical to allowing increments in experiment scale. Achievements over the past three years have more or less continued this pace; for example, combinatorial fluidic preindexing has increased the throughput of droplet-based single-cell RNA sequencing up to 15-fold. Figure adapted from references [133, 163].

**Box 1:**
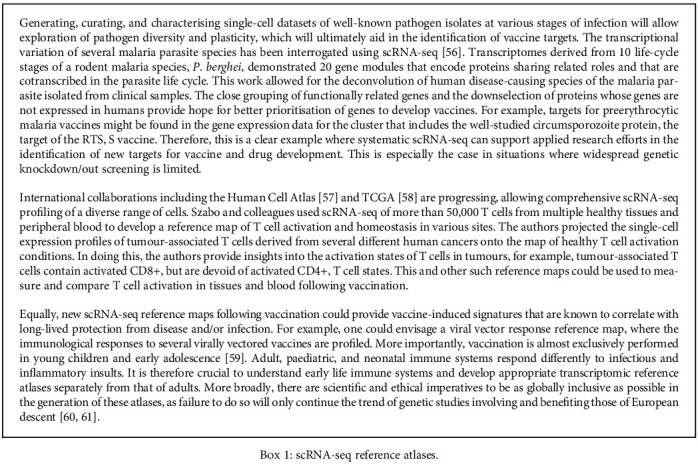
Box 1: scRNA-seq reference atlases.

**Box 2:**
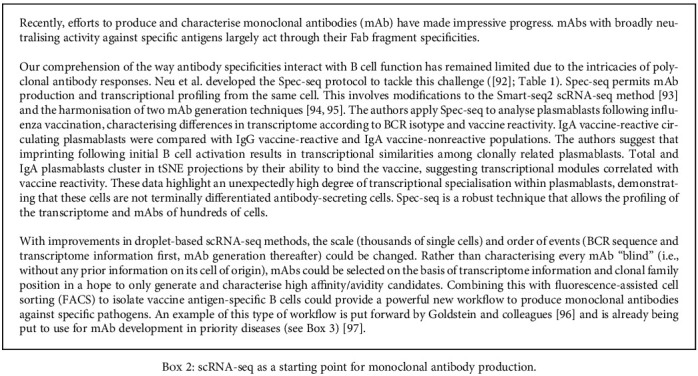
Box 2: scRNA-seq as a starting point for monoclonal antibody production.

**Box 3:**
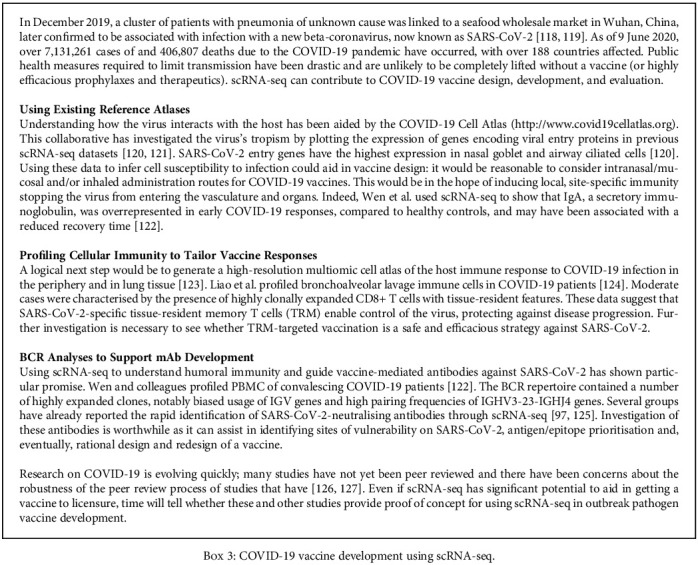
Box 3: COVID-19 vaccine development using scRNA-seq.

**Box 4:**
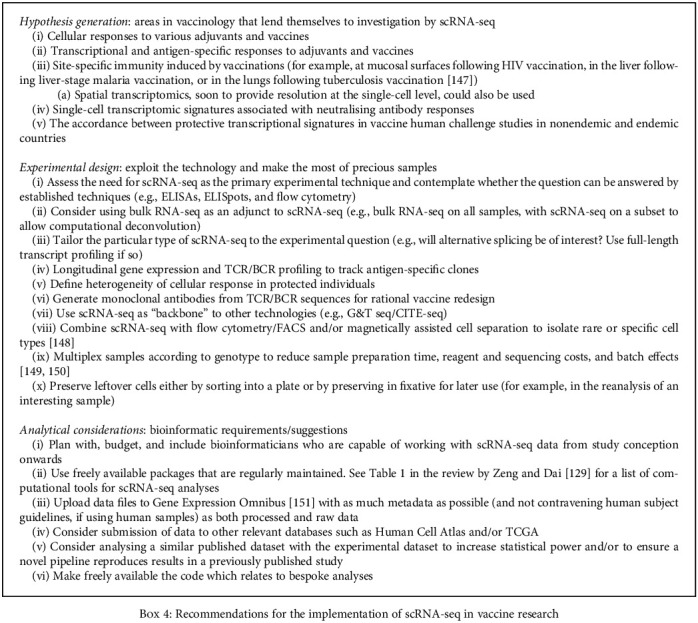
Box 4: Recommendations for the implementation of scRNA-seq in vaccine research

**Table 1:** Summary of prophylactic vaccinology publications using scRNA-seq.

Reference	Cell type	Species	Vaccine pathogen	scRNA-seq method
Afik et al. 2017. *Nucleic Acids Res.* [78]	CD8+ T cells	Human	Yellow fever	Smart-seq
Upadhyay et al. 2018. *Genome Med.* [80]	Plasmablasts	Human; Rhesus	Influenza; SIV	Smart-seq
Neu et al. 2019. *J Clin Invest*. [92]	Plasmablasts	Human	Influenza	Smart-seq/Spec-Seq
Cirelli et al. 2019. *Cell*. [81]	B cells	Rhesus	HIV	Smart-seq
Waickman et al. 2019. *Nat Commun.* [87]	CD8+ T cells	Human	Dengue	10X
Sheerin et al. 2019. *Sci Rep*. [63]	Neutrophils	Mouse	Neisseria meningitidis serogroup B	10X
Darrah et al. 2020. *Nature*. [133]	T cells	Rhesus	Tuberculosis	Seq-Well

## Appendix

### A. Experimental Considerations for scRNA-seq in Vaccinology

(1) Research question considerations
  (a) Ample consideration of the particular research question to be answered is required for any experimental design, but in particular scRNA-seq experiments
  (b) Potential questions that can be asked in scRNA-seq experiments
    - Does vaccination result in previously uncharacterised single-cell states?
    - What is the temporal sequence of cellular processes taking place after vaccination?
    - How does T cell and/or B cell clonal diversity change in response to vaccination?
    - What are the transcriptional differences among vaccine reactive/antigen-specific cells?
    - To what extent are vaccine-induced transcriptional changes reflected at the cellular (bulk vs. single-cell sequencing) and protein level (transcriptomics vs. proteomics)?
    - What are the single-cell transcriptional differences in vaccine response between vaccines A and B?
    - What is the single-cell transcriptional profile in peripheral lymphocytes (or organ) given a particular vaccine platform (e.g., virus-like particles), regardless of the antigen that is delivered?
  (c) Other specific experimental considerations will influence experimental design
    (i) Breadth vs. depth
      1. Are lowly expressed genes of particular interest? For transcripts that are lowly expressed (e.g., transcription factors), full length sequencing approaches may be better than 3′ methods [152]. In this case, the amount of sequencing should also be considered such that “saturation” is reached; that is, further sequencing does not lead to the discovery of more unique transcripts
      2. Are rare cell types to be profiled? Increasing cell number and maintaining read depth relatively low allows more power to detect rare cell populations (that may exist at <1% in frequency) [5]. It has been suggested that to estimate several important gene characteristics, the most favourable sequencing depth is around one read per cell per gene [153]. Alternatively, enrich the cell type of interest by using FACS, followed by scRNA-seq and use methods such as GateID to predict “nonintuitive” gating strategies based on scRNA-seq data [154]
    (ii) Will comparisons be made between different conditions (e.g., prime alone vs. prime-boost)?
    (iii) What are the qualities and expression levels of the marker genes of cell types of interest? Transcriptional bursting can result in substantially different transcript quantities and apparent gene expression levels [155]
    (iv) What is the overall budget of the project? Cost/cell profiled is an often-used metric for budgeting scRNA-seq experiments
    (v) What facilities and expertise are available?
    (vi) Has technical and experimental advice been sought by nonconventional means (consider the active scRNA-seq community on Twitter (particularly #scRNAseq and #scQA), ResearchGate, medRxiv, bioRxiv, EMBI-EBI training (https://www.ebi.ac.uk/training), and the Galaxy platform (https://usegalaxy.org/))
(2) Sample preparation and processing considerations
  (a) Processing samples up to the point where scRNA-seq can be performed is a process that can greatly affect the outcome of the experiment
    - Are there unchangeable constraints on sample collection/processing times? What effects, if any, will these have on the results?
    - Do samples have to be processed immediately or is there a window where gene expression will not be affected, without specific preservation?
    - Will the samples be preserved (e.g., flash frozen, fresh frozen, or formalin-fixation and paraffin-embedding) [156]?
  (b) scRNA-seq usually requires mechanical or enzymatic dissociation of samples to produce a single-cell suspension. Certain factors will affect this process
    - How fragile/robust are the samples?
    - How well is the tissue dissociated?
    - Which single-cell isolation procedure will be used (e.g., microdissection, reverse emulsion droplets, FACS into plates, and/or nanowell isolation)?
    - Are there preexisting protocols in the published literature that describe isolation techniques from the sample of interest (consider published, preprint, and commercial (e.g., 10x Genomics technical notes) literature)?
    - Will the cells be preserved (e.g., cryopreservation using DMSO, methanol fixation, or storage in commercially available formulations to preserve cells and their RNA) [157]?
    - Can a method be used that does not dissociate tissue (e.g., Slide-seq) as a baseline to check for dissociation effects? This will likely add extra cost and require additional technical expertise
  (c) Once cells (or nuclei) are in a suspension, the starting point for RNA capture is achieved
    - Which is more apt and feasible to answer your specific research question, nuclei or cells isolated from the sample [134]? Nuclei-derived transcripts have a higher intronic/exonic read ratio as they contain proportionally more pre-mRNAs and there is potential that a narrower time period is profiled as mRNAs in the cytoplasm may have existed for longer
    - What are the characteristics of the cells of interest (e.g., size and adherability)? Volume can vary widely cell-to-cell, this affects the absolute number of transcripts and can be reflected in the detected number of genes per cell [158]. Adherability of a cell may affect processes before sequencing, such as FACS
    - What quantity of input material (cells or nuclei) is there?
  (d) Assessing RNA quality. Before embarking on costly library preparation for every sample, it is important to ensure that the RNA has not significantly degraded:
    - RNA quality is typically measured using the RNA Integrity Number (RIN) algorithm [159]. This test is performed by isolating RNA from a sample of interest (typically bulk or 10s-100s of cells) and performing RNA microcapillary electrophoresis. The algorithm uses multiple features from the resultant electropherogram trace to score quality of the RNA from 1 (most degraded) to 10 (least degraded-highest integrity)
    - Is there enough of the sample to produce a RIN score test on each sample? If the site, conditions, or timing of sample collections is variable, it may lead to differences in RNA degradation between samples (and batch effects, discussed below). If a RIN score step can be built into each experiment before embarking on library preparation, then it can prevent spending money on low-quality samples
(3) Replicates, scale, sequencing, and batch considerations
  (a) Batch effects
    (i) Batch effects are random technical artefacts which occur during handling/processing. If batches correspond to different biological conditions, then it is largely impossible to determine what differences are biological vs. artefacts
    (ii) Avoid batch effects by
      1. sorting cells from different biological conditions into different wells of the same plate
      2. using genetic variants to post hoc assign sequenced cells back to their genetically unique donor
      3. using the expression of an inserted genetic construct (not recommended)
      4. using barcoded antibodies (Cell Hashing) to label samples after dissociation, but before cell-capture step, to multiplex samples [13, 160]
    (iii) Batch effects may be corrected by a number of bioinformatics tools and/or packages [161, 162]
  (b) Experiment scale
    - How many cells will be tested largely depends on the level of heterogeneity of the sample and on the number of available cells
    - Plate-sorted single cells are limited in the amount that can be handled compared to microfluidic platforms, which enable studies with several thousands of cells (Figure 1)
  (c) Method of amplification
    - Either exponential PCR-based amplification or linear in vitro transcription (IVT) amplification is usually used. IVT incorporates less PCR bias and erroneous bias as it is based on an unamplified RNA template [164]
  (d) Transcript position
    - Some protocols provide full-length transcript data, whereas others amplify only the 3′ or 5′ ends of the transcripts
    - Non-full-length transcript methods allow increased throughput of cells, while full-length transcripts are advantageous if splice variants are important, looking to detect genetic variants or when studying species that have poorly annotated genomes
  (e) Sensitivity
    - This is the ability of an assay to capture an mRNA molecule from a single cell within the final library. Low sensitivity protocols have a disproportionate effect on weakly expressed genes (e.g., genes encoding cytokines)
    - If weakly expressed genes are to be evaluated, consider higher sensitivity methods or consider “clean-up” procedures such as rRNA removal [165]
  (f) Ultimately, the particular protocol must be decided on an individual experiment basis. It is also important to note that while scRNA-seq methods have greater sensitivity than bulk RNA-seq methods, bulk methods have higher accuracy [166]

### B. Review Search Strategy

We performed an initial scoping review of the literature using MEDLINE/PubMed to identify major themes present in the vaccinology literature. Thereafter, we performed predefined searches in the context of the vaccinology themes we identified: controlled human infection studies, correlates of protection, profiling the immune response to infection, cancer vaccines, understanding host-pathogen interactions, comparing vaccine regimens and responses, adjuvants, animals as models for human diseases and natural infections of livestock, antigen screening/selection, and effects of coinfection on vaccination. Predefined searches included using MeSH terms: “Sequence Analysis, RNA”, “Vaccinology”, “Vaccines.” In January and February 2020, we searched for published literature in MEDLINE/PubMed and Embase and for preprint/not yet peer-reviewed literature in bioRxiv, medRxiv, Wellcome Open Research and arXiv. Further searches were conducted using free text to expand our capture of scRNA-seq (e.g., (“scRNA-seq” OR “single cell sequencing” OR “single cell RNA sequencing”)), vaccine (e.g., (vaccin∗ OR immunis∗ OR immuniz∗)) and theme-specific studies (e.g., (“vaccine correlate∗” OR “correlate of protection” OR “correlates” OR “correlate of vaccine protection”)).

After performing the searches, we refined the scope of our review to include only scRNA-seq studies—as opposed to those exclusively considering bulk RNA-seq—performed in humans or animal models of human disease published after 2015, unless they were considered sufficiently relevant to the narrative of our review.
